# Progress in Surgery of the Stomach

**Published:** 1903-01-24

**Authors:** 


### PROGRESS IN SURGERY OF THE STOMACH.

**Gastrostomy.**—Tenier and Gosset[1] advocate the direct method of gastrostomy. They incise the abdominal wall internal to the left linea semilunaris above the umbilicus; divide the anterior sheath of the rectus muscle; split the muscle; divide the posterior sheath, and so open the peritoneum. The stomach caught forward by four pressure forceps is drawn out and stitched in the following layers:—(1) Through the musculo-serous layer to the peritoneum and posterior sheath of the rectus; (2) through the musculo-serous layer of the stomach to the anterior sheath of the rectus muscle; (3) the musculo-serous layer is then transfixed, and through the opening thus made the mucous coat is drawn up, transfixed, and the edges sutured to the skin. The rest of the incision is then sutured. The opening goes directly into the stomach, the muscle acts as a sphincter, and the bulging mucous membrane as a plug. Of eight cases so treated one died in 24 hours, four lived 17 to 41 days, and one was alive nine months after operation, one four months, and one three months after the gastrostomy. They consider that the criterion of efficiency in gastrostomy is the continuance of the opening, and this they maintain is guaranteed by the plug of mucous membrane.

**Gastro-Enterostomy.**—Dalziel[2] says that of the utility of this operation in pyloric obstruction, simple or malignant, there can be no doubt whatever, and much benefit can be obtained in cases where the normal action of the stomach is prevented, or at least materially retarded, by adhesions, either in the region of the pylorus or over the general surface of the organ, due, as in many cases to plastic peritonitis from a perforating gastric ulcer, or to pathological changes in the neighbouring viscera. It is probable, also, that in another group of cases due merely to functional disorder, where we have dilatation of the organ without obstruction at the outlet, but accompanied by the usual fermentative changes, we have in gastro-enterostomy a means of draining the organ, and thereby alleviating the misery which has been found to be unrelieved by any ordinary method of treatment. Doubtless the operation is most useful and most frequently practised in those cases where there are marked organic causes leading to the dilatation. He reports 30 operations undertaken for the cure of apparently incurable dyspepsia, many of them with evidence of marked pyloric obstruction. Only one of these cases died.

**Gastro-jejunostomy.**—Hall[3] suggests a new route for this operation, and gives notes of four cases. He says the ordinary operation of posterior gastro-jejunostomy (through the transverse mesocolon) has failed to become universally adopted on account of certain disadvantages connected with the route by which the anastomosis is effected. It cannot be performed outside the body cavity in the manner that the anterior operation can, and by thus involving a greater peritoneal exposure it increases the risk of shock and peritonitis. Moreover, the necessary perforation of the transverse mesocolon may occasion troublesome hæmorrhage from mesenteric veins. Granted, therefore, that a posterior opening in the stomach has advantages, it remains a question whether the objections to the usual posterior route do not outweigh them. The author has noticed how easily the posterior wall of the stomach can be reached through the gastro-colic omentum, and he thinks that this route avoids the objections which have been urged against the usual posterior operation. The gastro-colic omentum is a thin, functionless and practically bloodless membrane, through which the finger can be easily pushed, and is readily accessible through a small abdominal incision. Moreover, when the stomach is dilated, its posterior wall sags downwards below the line of its greater curvature in such a way that it can be drawn through an opening in this omentum and the whole procedure performed outside the incision. A posterior anastomosis is thus obtained with a minimum of peritoneal exposure and without interfering with the transverse meso-colon. The jejunum is drawn up in front of the transverse colon, as in the anterior operation, but the author is not aware that that is any disadvantage. It usually crosses the colon well to the left where the latter passes backwards to the splenic flexure, and consequently no pressure is exercised upon it.

[1] Scottish Med. and Surg. Jour., June 1902, p. 548. [2] Brit. Med. Jour., Nov. 8, 1902, p. 1505. [3] Lancet, Sept. 6, 1902, p. 657.

*(To be continued.)*

